# Bottom Plate Damage Localization Method for Storage Tanks Based on Bottom Plate-Wall Plate Synergy

**DOI:** 10.3390/s25082515

**Published:** 2025-04-16

**Authors:** Yunxiu Ma, Linzhi Hu, Yuxuan Dong, Lei Chen, Gang Liu

**Affiliations:** 1 College of Pipeline and Civil Engineering, China University of Petroleum (East China), Qingdao 266580, China; yunxiu611@126.com (Y.M.); hulz22@163.com (L.H.); b23060018@s.upc.edu.cn (Y.D.); liugang@upc.edu.cn (G.L.); 2 Pipe-Network Group (Xuzhou) Pipeline Inspection and Testing Co., Xuzhou 221000, China

**Keywords:** t-shaped structure, ultrasonic guided waves, damage detection, total focusing imaging (TFM), collaborative bottom plate-wall plate detection

## Abstract

Ultrasonic guided waves can be employed for in-service defect detection in storage tank bottom plates; however, conventional single-array approaches face challenges from boundary scattering noise at side connection welds. This study proposes a collaborative bottom plate-wall plate detection methodology to address these limitations. Sensor arrays were strategically deployed on both the bottom plate and wall plate, achieving multidimensional signal acquisition through bottom plate array excitation and dual-array reception from both the bottom plate and tank wall. A correlation coefficient-based matching algorithm was developed to distinguish damage echoes from weld-induced scattering noise by exploiting path-dependent signal variations between the two arrays. The investigation revealed that guided wave signals processed through data matching effectively preserved damage echo signals while substantially attenuating boundary scattering signals. Building upon these findings, correlation matching was implemented on guided wave signals received by corresponding array elements from both the bottom plate and wall plate, followed by total focusing imaging (TFM) using the processed signals. Results demonstrate that the collaborative bottom plate-wall plate detection imaging cloud maps, after implementing signal correlation matching, effectively suppress artifacts compared with imaging results obtained solely from bottom plate arrays. The maximum relative localization error was measured as 5.4%, indicating superior detection accuracy.

## 1. Introduction

Storage tanks, as critical storage equipment in the petrochemical industry, are prone to localized corrosion during their long-term service due to factors such as construction quality, erosion from stored media, and surrounding environmental conditions [1,2,3]. Consequently, there is an urgent need for an online inspection technology capable of rapidly locating defects in the tank floor.

Traditional non-destructive testing (NDT) techniques are mostly invasive, characterized by low efficiency and high consumption of human and material resources [4,5,6,7]. Ultrasonic guided waves, as a type of mechanical elastic wave constrained and guided by structural boundaries [8,9,10,11], exhibit excellent detection capabilities for both surface and internal defects. Moreover, guided waves in different modes demonstrate unique propagation characteristics due to their specific wave structures [12,13], leading to their widespread application in NDT [14,15,16]. Fakih et al. [17] conducted experimental and finite element studies on defects in friction stir welds and proposed an adaptive noise-assisted empirical mode decomposition (EMD) data processing method. This method evaluates the extent of damage to welds based on variations in captured signal replicas. Zhang et al. [18] achieved detection of a 5 mm × 1 mm crack defect behind a “T”-shaped pipeline support structure by exciting the CSH_0_ mode guided waves using electromagnetic transducers. Wu et al. [19] investigated a Rayleigh-type wave (RTW) characteristic guided wave in “T”-shaped welded joints and achieved long-distance detection of small defects in the “T”-shaped weld joint area by exciting this modal guided wave at the weld end face. Raišutis et al. [20] combined transmission tomography with Lamb waves excited on the outer side of the tank bottom plate, enabling damage detection within the tank floor. Lowe et al. [21] investigated the propagation characteristics of Lamb and SH_0_ mode guided waves in the bottom plate and wall plate of storage tanks through finite element modeling and experimental validation. Their findings revealed that SH_0_ mode guided waves exhibit a higher signal-to-noise ratio (SNR) during tank inspections. Wang et al. [22] developed a magnetostrictive SH_0_ mode electromagnetic transducer for tank bottom plates and implemented a rotational focusing imaging method, achieving multi-defect detection within the inspection area.

**Figure 1:**
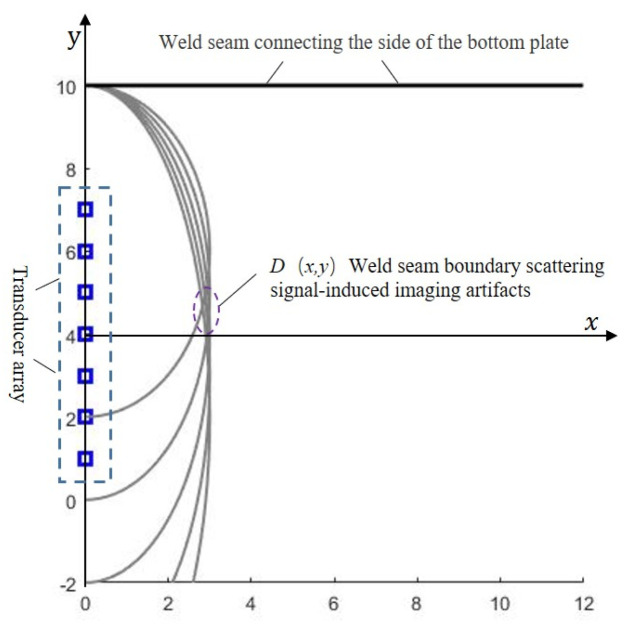
Schematic illustration of interference effects caused by side connection welds on the storage tank bottom plate.

In damage imaging processes, high-quality defect visualization and accurate localization can typically be achieved when the input signals exhibit a high signal-to-noise ratio (SNR). However, in large-scale containment structures such as petroleum storage tanks, the bottom plate assemblies fabricated through welding of multiple steel plates inevitably contain heterogeneous welded joints (e.g., butt welds and lap joints) with varying geometric configurations. During non-destructive testing (NDT) of the outermost edge plates in tank bottom structures, the discontinuous boundary scattering echoes caused by geometric and material discontinuities at sidewall weld connections are practically unavoidable, as illustrated in Figure 1. These scattering signals, when focused through transducer arrays, generate ghost echoes resembling structural artifacts within the inspection zone.

**Figure 2:**
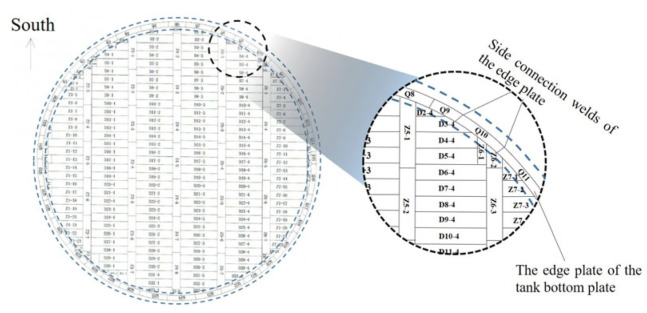
Schematic of the storage tank bottom plate structure.

Figure 2 delineates the peripheral edge plate arrangement. Conventional guided wave testing methodologies position transducers on these exterior edge plates, leveraging wave transmission through wall structures for internal defect detection. The inevitable presence of ambient noise during field inspections introduces positioning inaccuracies in damage imaging, rendering single-modal detection techniques insufficient for reliable assessment. Consequently, recent advancements focus on boundary scattering mitigation strategies [23,24,25]. Yu et al. [26] developed a wavelet reconstruction time-reversal (WR-TR) method integrating multimodal superposition finite-difference time-domain (MS-FDTD) simulations with maximum energy frame (MEF) analysis, achieving ≤2.3 mm localization accuracy for plate defects under strong boundary scattering interference. Huang et al. [27] innovatively treated boundary-reflected signals as virtual excitation sources, enabling composite plate defect detection through probabilistic damage imaging. Hall et al. [28] proposed sparse-array multipath guided wave imaging utilizing structural reverberations to construct signal dictionaries, validated on both aluminum and composite plates. Zhang et al. [29] proposed a multipath edge-reflected defect imaging methodology. This approach employs mirror points from four edges of a plate structure as additional virtual transmitters and receivers. By systematically identifying all potential propagation paths of edge-reflected wave packets, the method detects defect-induced damage paths through comprehensive path tracing. The final imaging synthesis is achieved through a multiplicative fusion strategy, which effectively integrates the multi-path detection results. This innovative technique enables reliable damage detection in conventional blind zones that are inaccessible to traditional inspection methods.

This paper addresses the phenomenon of artifacts caused by the reflection signals of guided waves from the side connection welds during array-based detection of the tank bottom. A collaborative detection method for the tank bottom and wall plate, tailored to the special “T”-shaped structure of the tank bottom, is proposed. By arranging transducer arrays on both the tank bottom and the wall plate, multidimensional data matrices from both components are acquired. Based on the differences in the propagation paths of boundary scattering echoes to the tank bottom and wall plate, the information received from both components is matched and fused. This approach effectively reduces the impact of boundary scattering signals and ultimately achieves defect detection behind the “T”-shaped structure of the tank bottom using the total focusing imaging method.

## 2. Dispersion Curve Construction for Storage Tank Baseplate

**Figure 3:**
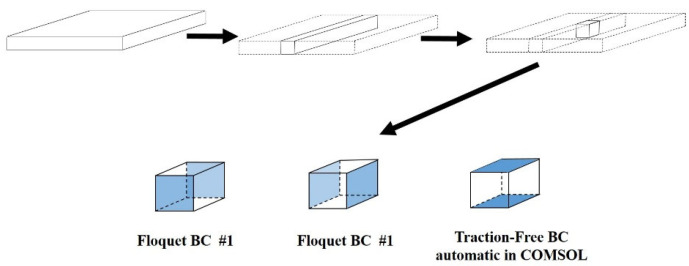
Schematic of the unit cell model implemented in the Floquet boundary condition (Floquet BC) method.

The Wave Finite Element Method (WFE), a numerical approach specifically designed for wave propagation analysis, combines analytical wave propagation principles with conventional finite element advantages. This method effectively addresses dynamic problems in periodic structures and gradually varying non-uniform configurations. By utilizing established commercial software to model only periodic substructures, WFE achieves full waveguide analysis while substantially reducing model dimensions and computational expenses compared to finite element eigenvalue frequency approaches. The Floquet Boundary Condition (Floquet BC) method [30], originally developed for deriving dispersion curves of periodic waveguides, embeds periodicity characteristics into computations, thereby limiting solutions to a single unit cell and simplifying problem complexity. See Figure 3.

Numerical simulations were conducted using the COMSOL Multiphysics 6.1 Solid Mechanics module, where a 0.2 mm × 0.2 mm × 20 mm unit cell of the storage tank baseplate was modeled. A free tetrahedral meshing strategy was implemented with strictly identical mesh configurations on periodic boundary interfaces, employing the predefined “Extremely fine” mesh size setting to ensure precision.

**Figure 4:**
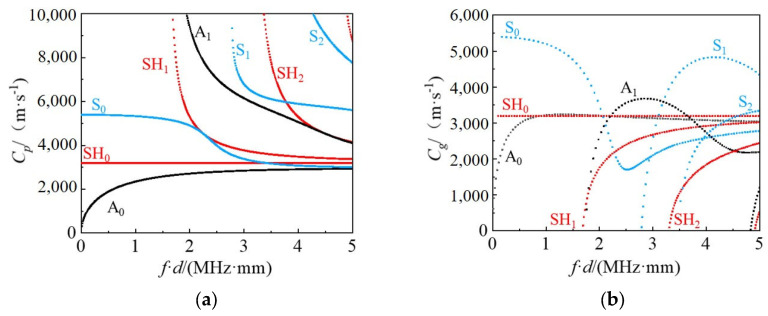
Dispersion characteristics of storage tank bottom plate structure (**a**) Phase velocity spectrum (**b**) Group velocity profile.

The materials used for both the tank bottom plate and wall plate are Q235 carbon steel, with an elastic modulus of 208 GPa, a density of 7850 kg/m^3^, and a Poisson’s ratio of 0.3. By employing the wave finite element method, the dispersion curves of guided waves in the tank bottom plate and wall plate structure were calculated. The results are shown in Figure 4 below:

From the dispersion curves of the tank bottom plate shown in Figure 4, it can be observed that, compared to the A_0_ and S_0_ mode Lamb waves, the SH_0_ mode guided waves maintain a stable propagation velocity across different frequency-thickness products due to their unique vibration propagation characteristics. This stability makes SH_0_ mode guided waves particularly ideal for damage detection applications. Therefore, this study will focus on the stable propagation characteristics of SH_0_ mode guided waves to conduct further investigations for damage detection.

## 3. Experimental Setup

**Figure 5:**
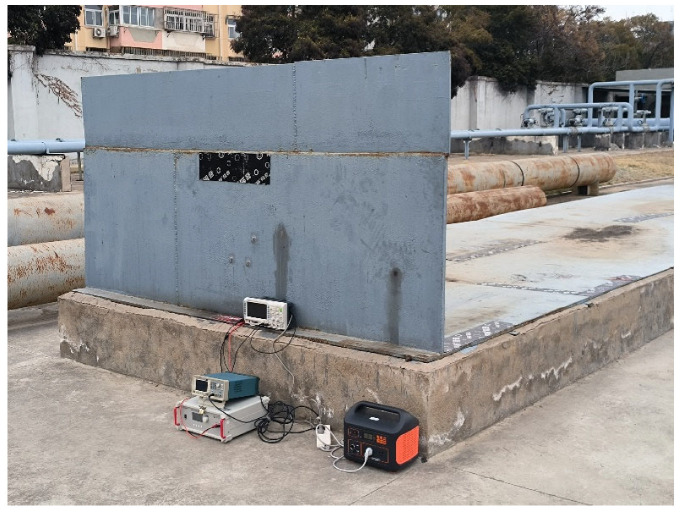
Experimental setup diagram of the “T”-shaped structure of the tank bottom plate.

The “T”-shaped structure in the experimental setup represents a 100,000-cubic-meter tank bottom model. Both the bottom plate and wall plate are made of Q235 structural carbon steel. The thickness of the bottom plate is 20 mm, while the wall plate has a thickness of 30 mm. The length of the edge plate on the outer side of the tank is 120 mm, and the overall width of the bottom plate is 3 m. The specific experimental platform is shown in Figure 5. In this study, the signal generator used is a Tektronix AFG1062 function generator (Tektronix, Beaverton, OR, USA), which is capable of effectively generating sine waves, square waves, and arbitrary waveforms. During the experiment, the guided wave signal excited is a five-cycle Hanning window-modulated sine wave with a center frequency of 65 kHz. The specific modulation method is shown in Equation (1). The signal amplifier is an ATA-2021H (Aigtek, Xi’an, China), with a controllable voltage gain of up to 60 times and a maximum output voltage of 200 V. The oscilloscope is a RIGOL DS1104 (RIGOL, Suzhou, China), which supports multi-channel data acquisition with a maximum real-time sampling rate of 1 GSa/s in full-channel mode. In this experiment, the sampling rate of the oscilloscope is set to 12.5 MSa/s. The transducers used are d15-type 10 × 10 × 3 mm piezoelectric shear plates, which can effectively excite SH_0_ mode guided waves when excited perpendicular to the polarization direction of the transducer. The coupling agent used is a metal adhesive, which exhibits excellent compatibility between ceramics and metals. Additionally, it possesses fast-drying properties, allowing for rapid and high-strength coupling of the transducer with the wall plate.

Hanning window function expression:

(1) $$A_{t}=\left\{\begin{array}{c} u(t)=0.5\left[1-cos(2\pi f\times \frac{t}{n})\right]\cdot sin(2\pi ft),0\leq t\leq n/f\\ 0,t\geq n/f \end{array}\right.$$

where *f* represents the central frequency of the signal in kHz; *t* is the time interval in seconds (s); *n* denotes the number of periods of the signal.

## 4. Damage Localization Methodology

### 4.1. Total Focusing Imaging-Based Localization Using Bottom Plate Array

**Figure 6:**
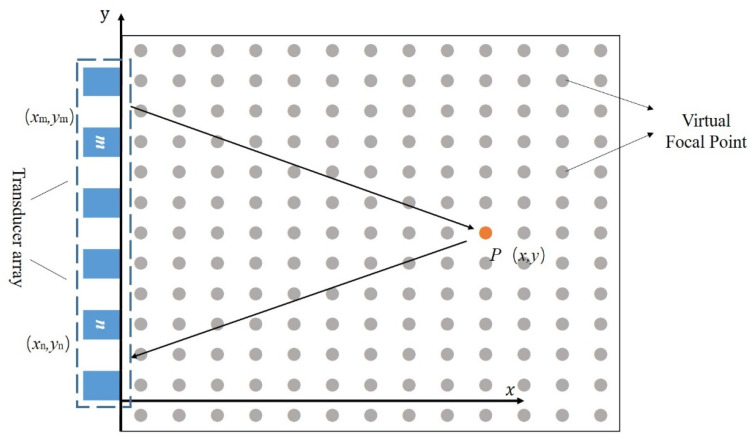
Schematic of the Total Focus Imaging Principle.

Total focusing method (TFM), as an advanced ultrasonic array inspection technique, involves sequential excitation and reception of array elements. During the data acquisition phase, it enables the collection of data from all excitation and reception channels within the array, providing a rich dataset for subsequent processing. The TFM algorithm divides the imaging area into a grid and calculates the acoustic propagation path between each array element and each grid point. Based on the group velocity of the guided waves, the corresponding time is computed, and the signal amplitude at that time in the acquired data is assigned to the corresponding grid point. This process is repeated iteratively, and the accumulated amplitude information is used as the grayscale value for the pixel, thereby achieving full-focus imaging of the region. The schematic diagram of the principle of Total Focus Imaging is shown in Figure 6 below.

During the online inspection of storage tank bottoms, non-invasive methods are typically employed, and the transducer array can only be positioned on the exterior of the tank. Therefore, the most straightforward approach in practical detection is to arrange the transducer array on the outer edge plate of the tank bottom. By exciting and receiving array elements, damage information from different regions of the bottom plate is collected, enabling the imaging and localization of damage defects on the bottom plate.

Taking the array inspection of the tank bottom as an example, consider a single propagation path involving an excitation element (*x*_m_, *y*_m_), a reception element (*x*_n_, *y*_n_), and a damage point *P* (*x*, *y*). The time-of-flight *T* (*x*, *y*) along the propagation path is calculated based on their geometric relationship.

(2) $$T(x,y)=\frac{\sqrt{{\left(x_{m}-x\right)}^{2}+{\left(y_{m}-y\right)}^{2}}+\sqrt{{\left(x_{n}-x\right)}^{2}+{\left(y_{n}-y\right)}^{2}}}{C_{g}}$$

where *x*_m_ represents the horizontal coordinate of the excitation transducer, in millimeters (mm); *y*_m_ denotes the vertical coordinate of the excitation transducer, in millimeters (mm); *x*_n_ is the horizontal coordinate of the receiving transducer, in millimeters (mm); *y*_n_ is the vertical coordinate of the receiving transducer, in millimeters (mm); *C*_g_ is the group velocity of the excited guided wave, in meters per second (m/s); *T* (*x*, *y*) is the time-of-flight of the guided wave signal from the excitation element to the receiving transducer after being reflected by the damage, in seconds (s).

After calculating the time-of-flight of the damage signal along the propagation path, the amplitude information of the corresponding echo is extracted. This amplitude information is assigned to the grid point at the corresponding distance. By sequentially superimposing these values, the final amplitude at the focal point is obtained, enabling the focused imaging of the damage signal.

(3) $$I(x,y)=\sum_{i=1}^{N}\sum_{j=1}^{N}S_{\mathrm{ij}}(T_{\mathrm{ij}}(x,y))$$

where *T*_ij_ represents the time taken for the guided wave excited by transducer *i* to be received by transducer *j*, in seconds (s); *S*_ij_ denotes the characteristic amplitude information extracted from the signal received by transducer *j* when excited by transducer *i*, in volts (V); and *I* (*x*, *y*) is the final amplitude at the focal point after superimposing the amplitude information from multiple damage paths, in volts (V).

### 4.2. Collaborative Bottom Plate-Wall Plate Damage Detection Methodology

When inspecting the bottom plate of a storage tank, leveraging its unique “T”-shaped structure, in addition to acquiring guided wave signals from the bottom plate for detection, a transducer array can also be deployed on the tank wall to obtain more comprehensive data. During the experiment, the transducers on the tank bottom are sequentially excited, and the signals are received by the transducers on both the bottom plate and the wall plate. This dual-array data acquisition from the bottom plate and wall plate enables the collection of multidimensional information.

In practical non-destructive testing processes, since the tank bottom plate is constructed by welding multiple steel plates, it is inevitable to receive non-damage echo noise signals reflected from the side connection welds of the bottom plate. These signals can undoubtedly introduce certain negative effects on the detection process. In this experiment, a d15-type piezoelectric shear transducer is used. When excited perpendicular to the polarization direction of the transducer, the SH_0_ mode is dominant, while excitation along the polarization direction generates the S_0_ mode. Due to the non-dispersive and mode-conversion-free propagation characteristics of the SH_0_ mode guided waves, they are utilized for damage detection in the tank bottom plate. During the detection process, the damage reflection signals within the detection area in front of the array are SH_0_ mode guided waves, whereas the boundary reflections are S_0_ mode guided waves.

When the guided wave signals propagate from the bottom plate to the tank wall, the plate thickness changes, causing the frequency-thickness product to shift from 1.3 MHz·mm to 1.95 MHz·mm. Due to the non-dispersive nature of the SH_0_ mode, its velocity remains unchanged. Additionally, since the damage echoes propagate the same distance to the bottom plate and the tank wall, the time-domain signals received by the bottom plate and the tank wall maintain high correlation. In contrast, the S_0_ mode guided waves propagating toward the side of the array undergo boundary reflection, and their wave packets propagate from both the bottom plate and the wall plate to the receiving array. When the frequency-thickness product of the S_0_ mode changes from 1.3 MHz·mm to 1.95 MHz·mm, its propagation speed decreases from 4970 m/s on the bottom plate to 3657 m/s on the wall plate. Due to this velocity difference, the time-domain discrepancy between the two signals increases with distance, leading to a corresponding reduction in their correlation.

**Figure 7:**
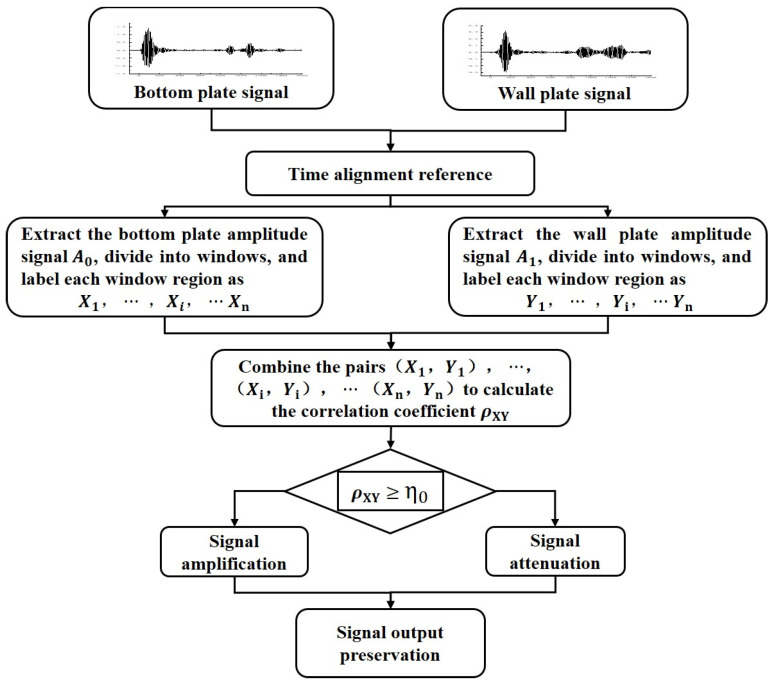
Flowchart of the correlation-based processing workflow for guided wave signals from the bottom plate and wall plate.

Therefore, based on the differences in the propagation paths of damage echoes and boundary-scattered noise signals to the bottom plate and wall plate arrays, a data processing method involving mutual matching and calibration of signals from the bottom plate and wall plate is proposed. By extracting the guided wave signals from the corresponding array elements on the bottom plate and wall plate, and aligning the initial reference points of the signals, the amplitude signals of the bottom plate (*A*_0_) and the wall plate (*A*_1_) are obtained. The amplitude signals from the bottom plate and wall plate are divided into multiple windows, and the correlation coefficient $\rho_{\mathrm{XY}}$ between the signals from the bottom plate and wall plate is calculated sequentially using a sliding window approach combined with Equation (4). The correlation between the two waveform signals is determined based on the magnitude of the correlation coefficient in the corresponding window. During this process, a predefined correlation coefficient threshold $\eta_{0}$, combined with Equation (5), is used to enhance the parts of the bottom plate signal with high correlation and attenuate those with low correlation. The flowchart of the correlation matching data processing is shown in Figure 7. After processing, the data are subjected to total focusing imaging, thereby achieving collaborative detection of the bottom plate and wall plate.

(4) $$\rho_{\mathrm{XY}}=\frac{Cov(A_{0},A_{1})}{\sigma_{0}\sigma_{1}}$$

where $\rho_{\mathrm{XY}}$ represents the correlation coefficient between the bottom plate and wall plate signals within the current window; $Cov(A_{0},A_{1})$ denotes the covariance between the amplitude signals of the bottom plate (*A*_0_) and wall plate (*A*_1_) within the current window; $\sigma_{0}$ and $\sigma_{1}$ are the standard deviations of the amplitude signals (*A*_0_ and *A*_1_, respectively) within the current window.

(5) $$A_{0}^{\prime }=\left[\begin{array}{l} A_{0}(1+\frac{\left|\rho_{\mathrm{XY}}-\eta_{0}\right|}{1-\eta_{0}}),\rho_{\mathrm{XY}}\geq \eta_{0}\\ A_{0}(1-\frac{\left|\rho_{\mathrm{XY}}-\eta_{0}\right|}{\eta_{0}}),\rho_{\mathrm{XY}}<\eta_{0} \end{array}\right.$$

where $A_{0}^{\prime }$ represents the processed amplitude signal of the tank bottom plate in the current window, in volts (V); $A_{0}$ denotes the raw amplitude signal of the tank bottom plate in the current window, in volts (V); $\rho_{\mathrm{XY}}$ is the correlation coefficient in the current window; $\eta_{0}$ is the correlation processing threshold.

**Figure 8:**
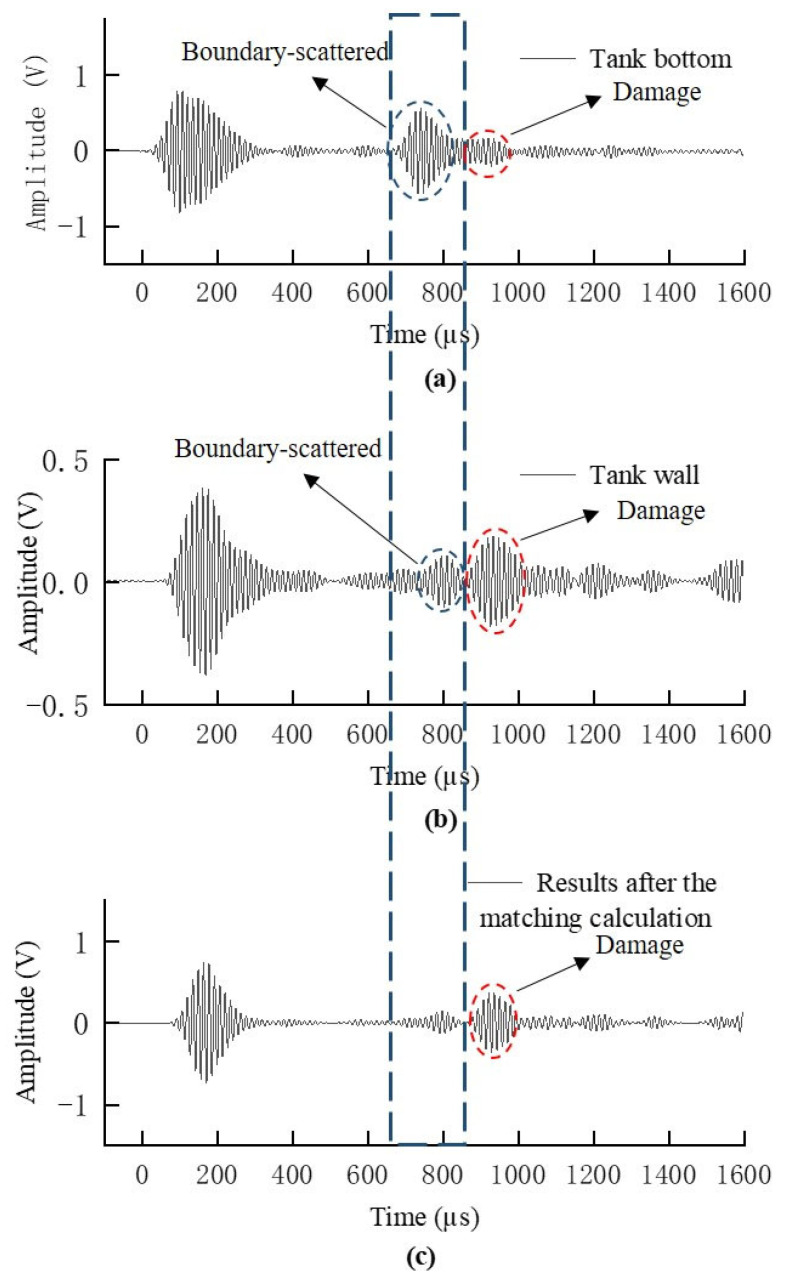
Signal correlation processing results for guided waves from the tank bottom plate and wall plate. (**a**) Detection waveform received by the tank bottom array element; (**b**) Detection waveform received by the tank wall array element; (**c**) Waveform obtained after the matching and fusion of the waveform data from the tank bottom and wall array elements.

Based on the theoretical analysis above, a correlation analysis between the bottom plate and the wall plate was conducted. The guided wave signals received by the transducers located at symmetrical positions relative to the large fillet weld on the outer bottom plate and wall plate of the storage tank in array $D_{1}$(1330, 120) were acquired. The guided wave signals received by the bottom plate and wall plate of the storage tank were processed using the aforementioned correlation matching method. After optimization, the data processing window size was set to 100 data points per window, and the correlation processing threshold was set to 0.7. The results of the data processing are shown in Figure 8 below:

As shown in Figure 8 above, the time-domain detection signals of the storage tank bottom plate, wall plate, and the bottom plate after correlation matching processing are displayed. From Figure 8a,b, it can be observed that the damage echo signals on the bottom plate and wall plate, due to the non-dispersive nature of the SH_0_ mode guided waves, exhibit consistent time-of-flight when propagating the same distance to the bottom plate and wall plate. In contrast, the boundary-scattered S_0_ mode guided waves propagate partially along the bottom plate and are received by the bottom plate array, while the other portion propagates along the wall plate and is received by the wall plate array. Due to the difference in thickness between the bottom plate and the wall plate, the frequency-thickness product of the guided waves changes along different propagation paths, leading to variations in the group velocity of the boundary-reflected guided waves on the bottom plate and wall plate. Because of the differences in propagation paths and velocities of the boundary-scattered signals, the echo signals of the boundary scattering gradually diverge in the time domain between the bottom plate and the wall plate. The correlation between the boundary-scattered echo signals received by the bottom plate array and those received by the wall plate array progressively decreases. The results after correlation matching calculations are shown in Figure 8c, where the damage echo signals are well preserved, while the boundary-scattered echo signals are effectively attenuated.

## 5. Results and Analysis

### 5.1. Damage Detection via Bottom Plate Array Total Focusing Imaging

#### 5.1.1. Analysis of the Interference Effect of Side Boundary Scattering Signals on the Tank Bottom Damage Detection

**Figure 9:**
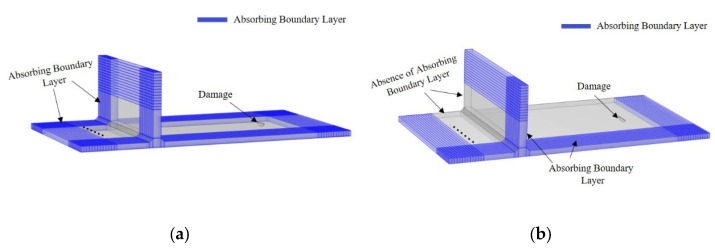
Finite element models for studying the influence of side connection welds on tank bottom plate detection. (**a**) Model without side connection weld reflections; (**b**) Model with side connection weld reflections.

To further validate the impact of scattering signals from the side welds of the storage tank bottom plate on the detection results during actual inspection processes, this study employs COMSOL 6.1 to establish a finite element model. In this model, the end-face scattering signals are used to simulate the influence of scattering signals from the side connection welds of the tank bottom plate during detection. A “T”-shaped structure model of the tank bottom plate, as shown in Figure 9, is constructed. The bottom plate thickness is set to 20 mm, and the tank wall thickness is set to 30 mm, consistent with the experimental setup. The material properties are set as shown in Table 1 below. A damage defect is placed 450 mm away from the array position. The SH_0_ mode guided wave is excited using a line source, and the guided wave signals are received using point probes. In this section, by modifying the boundary absorption layer of the finite element model, the influence of scattering signals from the side connection welds on the damage detection of the tank bottom plate is investigated.

To ensure the accuracy and stability of the solution process while reducing the computational data volume, the grid size should satisfy the spatial resolution criterion, i.e., the maximum grid size should be less than 1/6 of the minimum wavelength, and the time step should be less than 1/20 of the system’s highest response frequency. The specific calculation formulas are provided in (6) and (7).

(6) $$\Delta_{x,y,z}\leq \frac{\lambda }{6}$$

(7) $$\Delta t\leq \frac{1}{20f}$$

where, $\Delta_{x,y,z}$ denotes the tetrahedral element size (mm) in the finite element model, *λ* represents the minimum wavelength (mm) of the excited guided wave, Δ*t* indicates the computational time step (s), and *f* specifies the central frequency (kHz) of the excitation signal. This study employed a 65 kHz SH_0_ mode guided wave excitation. The maximum element size derived from Equation (6) was 8.1 × 10^−3^ m, while the maximum time step calculated via Equation (7) was 7.6 × 10^−7^ s. The global mesh size was set to 8.1 × 10^−3^ m, with local refinement zones at 4.05 × 10^−3^ m, using free tetrahedral mesh elements with a time step of 7.6 × 10^−7^ s.

This subsection investigates the influence of side-connection weld scattering signals on damage detection in storage tank bottom plates by modifying the absorbing boundary layer configuration in the finite element model. The ABLs adopt a multi-tiered configuration based on mesh gradation, with a total length L≥2λ and maximum absorption parameter $\alpha_{\max}=10f$. To mitigate acoustic impedance discontinuities, the thickness of the damping layers progressively increases from the inner to outer regions. The governing equations are provided in Equation (8).

(8) $$\alpha =\alpha_{\max}{\left(\frac{l}{L}\right)}^{n}$$

where, l denotes the length of each absorbing boundary layer (ABL) tier; L represents the total ABL length; a indicates the current-tier damping coefficient, while A corresponds to the maximum damping coefficient; *n* signifies the power exponent (assigned as 2).

**Figure 10:**
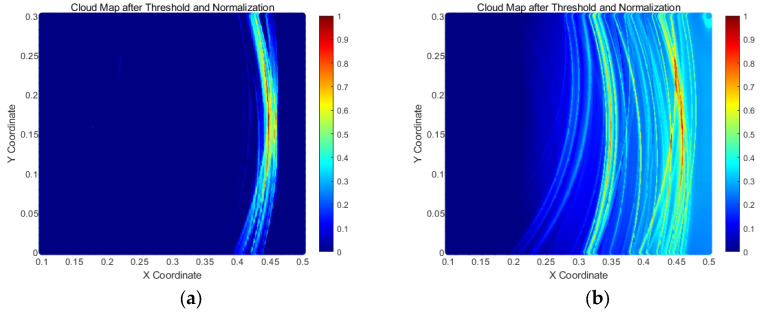
Total focusing imaging results from the bottom plate array in finite element models. (**a**) TFM results without interference from side connection weld reflections; (**b**) TFM results with interference from side connection weld reflections.

The TFM results of the signals received by the two model arrays are shown in Figure 10 below:

From the above finite element study, it is evident that under the interference of scattering signals from the side connection welds, the damage signal received by the bottom plate array can be focused and imaged at the 447 mm position. However, with the removal of one side’s absorbing boundary layer, Figure 10b shows that, in addition to the damage echo being focused and imaged at the 458 mm position, a non-damage artifact also appears at 351 mm. Furthermore, compared to Figure 10a, Figure 10b exhibits significantly more noise interference, which undoubtedly introduces a certain level of disturbance to the detection results.

#### 5.1.2. Damage Detection Experiment for Storage Tank Bottom Plate

In the detection of damage behind the “T”-shaped structure of the storage tank bottom plate, to simulate the actual online inspection process as closely as possible, neither the excitation transducer nor the receiving sensors can be placed inside the tank. The damage detection experiment was conducted by arranging a transducer array on the outer edge plate of the tank. Each array consists of ten transducers, positioned 100 mm from the large fillet weld, with a center-to-center spacing of 40 mm between each transducer element.

**Figure 11:**
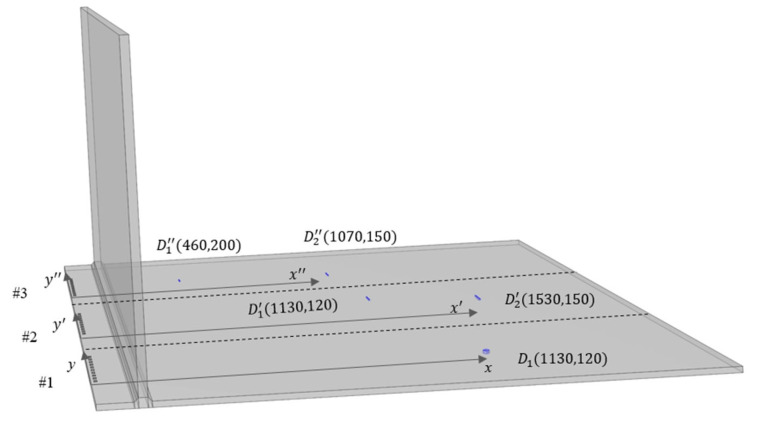
Schematic diagram of detection array and relative positions of damage defects on the tank bottom plate.

**Figure 12:**
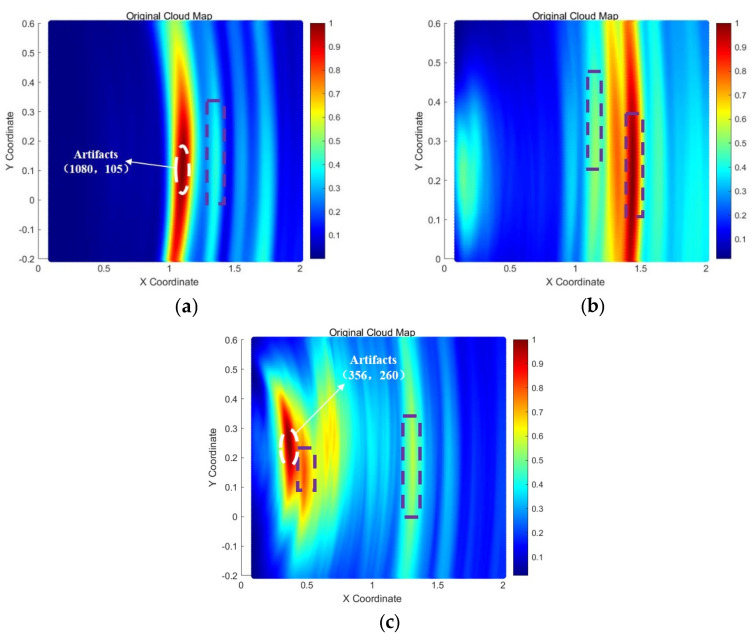
TFM cloud maps from bottom plate array detection. (**a**) Bottom plate array results for $D_{1}$ (1330, 120); (**b**) Bottom plate array results for ${D^{\prime }}_{1}$ (1130, 250) and ${D^{\prime }}_{2}$ (1530, 150); (**c**) Bottom plate array results for ${D^{\prime \prime }}_{1}$ (460, 200) and ${D^{\prime \prime }}_{2}$ (1070, 150).

In this study, three sets of experiments were conducted to detect damage on the storage tank bottom plate at different locations and distances. The arrangement of the transducer arrays was consistent with the description above. Taking the first transducer position from right to left as the origin, a planar coordinate system was established with the *y*-axis parallel to the large fillet weld and the *x*-axis perpendicular to the large fillet weld, extending radially inward along the tank bottom plate. The imaging area was divided into an 8 × 8 mm grid. Among the three sets of experiments, the first set involved detecting pitting corrosion, while the other two sets focused on crack defects. The position coordinates and dimensions of the defects are listed in Table 2 below:

The schematic diagrams of the three detection arrays and the locations of the damage defects are shown in Figure 11 below:

As shown in Figure 11, transducer arrays were arranged on the outer bottom plate of the storage tank for all three sets of parallel experiments. The data collected from the tank bottom plate were processed using the TFM, and the imaging results are presented in Figure 12 below:

In Figure 12, the purple squares represent the images of the damage defects. From the experimental results above, it is evident that by arranging a transducer array on the tank bottom plate and employing the total focusing method, defect signals can be successfully focused and imaged. However, due to the inevitable presence of boundary scattering signals from the bottom plate in the experimental environment, some unnecessary noise is introduced, leading to the appearance of artifacts in the images that do not correspond to actual defects. The most notable examples are the artifacts circled in white in Figure 12a,c, located at (1080, 105) and (356, 260), respectively. These artifacts exhibit significantly higher grayscale values than the actual damage defects, introducing strong interference and reducing the reliability of the detection results.

### 5.2. Collaborative Detection with Bottom Plate-Wall Plate Signal Fusion

**Figure 13:**
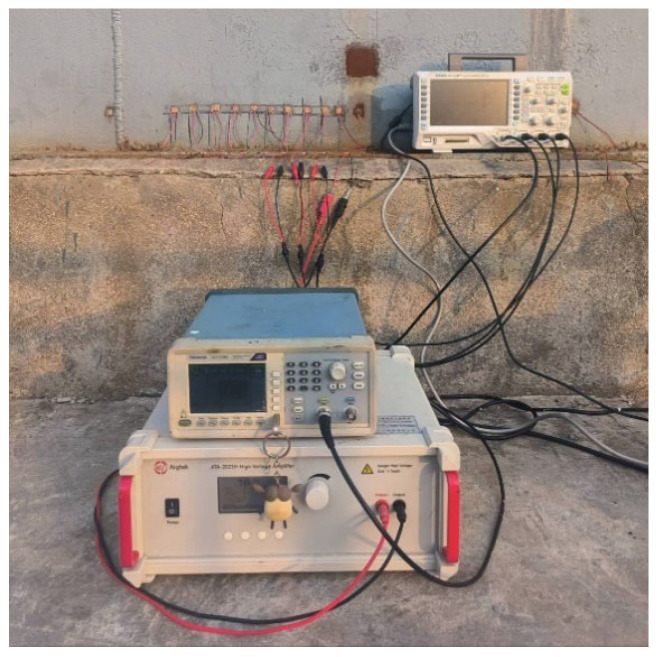
Experimental setup for collaborative bottom plate-wall plate damage detection.

**Figure 14:**
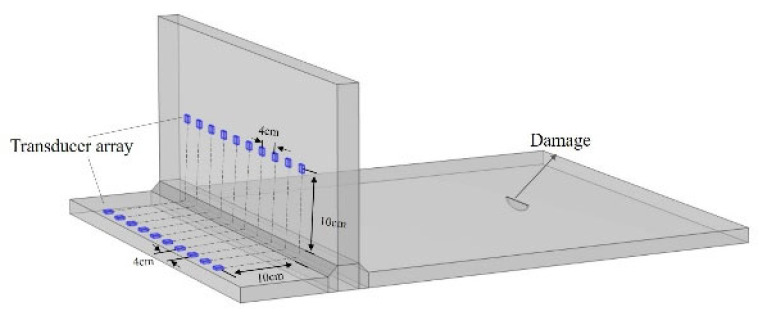
Schematic of collaborative bottom plate-wall plate damage detection.

Based on the theoretical findings above, it is demonstrated that using the correlation matching processing method for signals from the tank bottom plate and wall plate can effectively preserve damage echo signals while attenuating scattering signals caused by discontinuous interfaces. Therefore, the following section will incorporate this data processing approach to conduct collaborative bottom plate-wall plate detection experiments. Building on the bottom plate array described earlier, a wall plate transducer array is added, aligned parallel to the large fillet weld and the edge plate end face. The array elements on both the bottom plate and the wall plate are symmetrically positioned relative to the large fillet weld, with a distance of 10 cm from the weld and an inter-element spacing of 4 cm, as illustrated in Figure 13 and Figure 14. This setup is used to carry out collaborative detection research on the tank bottom plate and wall plate.

The signals from the bottom plate and wall plate transducers at symmetric positions relative to the large fillet weld were acquired. The signals received by the corresponding transducers were processed according to the method described in Figure 7, completing the matching of the bottom plate and wall plate signals. The time window and correlation threshold remained consistent with the previous settings. The processed data were then subjected to the total focusing imaging algorithm to achieve collaborative bottom plate-wall plate detection.

**Figure 15:**
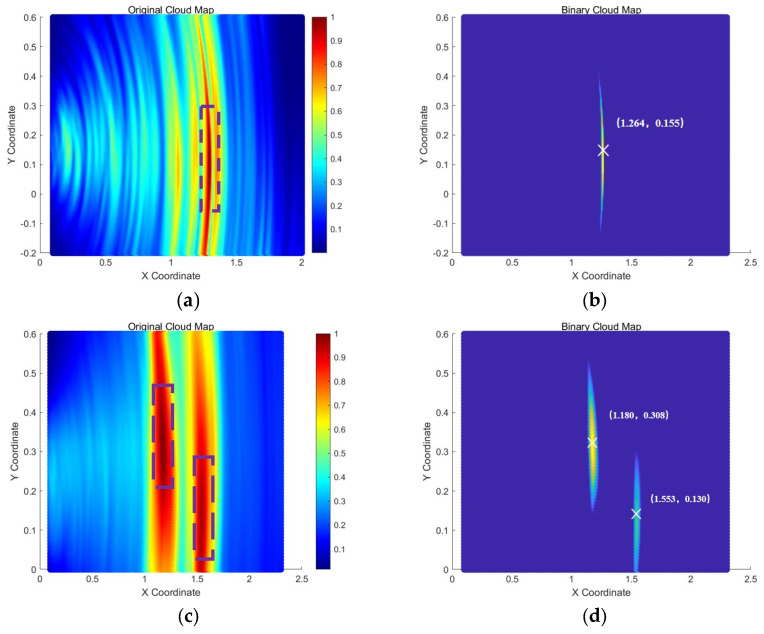

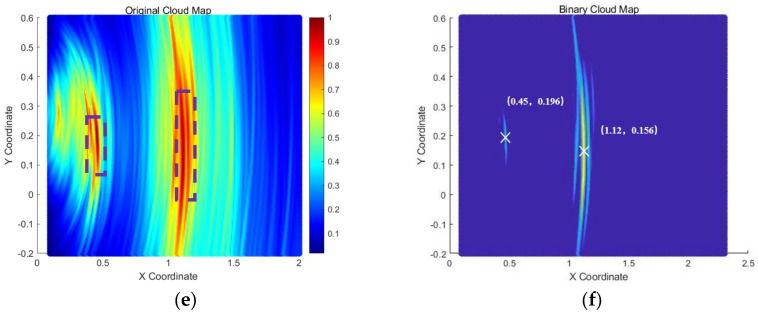
TFM cloud maps from collaborative bottom plate-wall plate array detection. (**a**) Collaborative results for $D_{1}$ (1330, 120); (**b**) Enhanced results for $D_{1}$ (1330, 120); (**c**) Collaborative results for ${D^{\prime }}_{1}$ (1130, 250) and ${D^{\prime }}_{2}$ (1530, 150); (**d**) Enhanced collaborative results for ${D^{\prime }}_{1}$ (1130, 250) and ${D^{\prime }}_{2}$ (1530, 150); (**e**) Collaborative results for ${D^{\prime \prime }}_{1}$ (460, 200) and ${D^{\prime \prime }}_{2}$ (1070, 150); (**f**) Enhanced collaborative localization results for ${D^{\prime \prime }}_{1}$ (460, 200) and ${D^{\prime \prime }}_{2}$ (1070, 150).

The imaging results of the collaborative bottom plate-wall plate detection are shown in Figure 15. The left-side images (a), (c), and (e) in Figure 15 represent the original imaging cloud maps of the collaborative detection results. To ensure the clarity and accuracy of damage identification, the pixel grayscale values in the left-side cloud maps that were less than 0.8 were set to zero, and the resulting damage localization cloud maps are shown in Figure 15b,d,f.

Comparing the imaging results in Figure 12 and Figure 15, the collaborative bottom plate-wall plate detection method effectively suppresses artifacts in the detection results compared to the independent imaging results obtained using only the bottom plate array. In all three parallel experiments, no artifacts with grayscale values higher than those of the actual damage defects were observed. In contrast to the single-array imaging results of the tank bottom plate, the collaborative bottom plate-wall plate detection method achieves clearer and more reliable localization and imaging of damage defects.

### 5.3. Error Analysis

In this experiment, 10 array transducers were arranged on the bottom plate and wall plate of the storage tank with a center-to-center spacing of 40 mm. Therefore, the central coordinate position of the detection array is (0, 220). The relative error was calculated by determining the distance ($r_{1}$) between the actual damage center coordinates and the array center coordinates, and the distance ($r_{2}$) between the detected damage coordinates and the array center coordinates. The specific calculation formula is as follows (9), (10) and (11).

(9) $$r_{1}=\sqrt{{\left(x_{1}-x_{0}\right)}^{2}+{\left(y_{1}-y_{0}\right)}^{2}}$$

(10) $$r_{2}=\sqrt{{\left(x_{2}-x_{0}\right)}^{2}+{\left(y_{2}-y_{0}\right)}^{2}}$$

(11) $$\delta =\frac{\left|r_{1}-r_{2}\right|}{r_{1}}\times 100\%$$

where (*x*_0_, *y*_0_) represents the central coordinate position of the transducer array in millimeters (mm); (*x*_1_, *y*_1_) denotes the central coordinate position of the actual damage location, and (*x*_2_, *y*_2_) represents the central coordinate position of the detected damage, both in millimeters (mm). $r_{1}$ and $r_{2}$ are the relative distances from the actual damage and the detected damage to the central coordinate of the transducer array, respectively, in millimeters (mm). δ is the relative error between the relative distance from the actual damage defect to the central coordinate of the transducer array and the relative distance from the detected defect coordinates to the central coordinate of the transducer array. The specific calculation results are shown in Table 3 below:

The damage detection experimental results for the tank bottom plate indicate that the damage localization method based on the proposed bottom plate-wall plate collaborative detection can successfully identify damage defects at different locations on the bottom plate. Compared to full-focus imaging with the bottom plate array, the bottom plate-wall plate collaborative detection method not only successfully detects damage at three different positions and distances on the bottom plate but also demonstrates significant effectiveness in suppressing artifacts caused by boundary reflections. In the three experimental cases, the artifact suppression rate was 100%. As shown in Table 3, the maximum relative error of damage localization using the total focusing imaging method with the bottom plate array is 15.3%, whereas the maximum relative error using the collaborative bottom plate-wall plate detection method is only 5.4%, indicating excellent detection accuracy.

Compared to the conventional bottom plate array detection method, the proposed collaborative bottom plate-wall plate detection method enhances the detection process by deploying transducer arrays on both the bottom plate and the wall plate, thereby acquiring multidimensional information about the tank bottom plate. By incorporating a correlation matching processing approach, the signals from corresponding array elements on the bottom plate and wall plate are matched, effectively reducing boundary scattering noise. This approach significantly suppresses the generation of artifacts during detection. In all three parallel experiments of tank bottom plate damage detection, no artifacts with grayscale values higher than those of the actual damage defects were observed, providing a more reliable detection outcome for tank bottom plate damage inspection.

## 6. Conclusions

The collaborative bottom plate-wall plate ultrasonic guided wave damage localization method proposed in this study is designed for T-shaped storage tank structures where conventional bottom plate array detection faces challenges such as: significant detection errors caused by overlapping wave packets from boundary scattering signals at side connection welds (${D^{\prime \prime }}_{2}$), and artifacts generated by the focusing of boundary scattering signals within the detection zone. To address these issues, transducer arrays are deployed on both the bottom plate and wall plate. By exciting the bottom plate and receiving signals simultaneously from both arrays, multidimensional information about the tank bottom plate is acquired. Guided wave signals from corresponding array elements on the bottom plate and wall plate are processed using a correlation coefficient matching algorithm to attenuate boundary scattering interference. Subsequent total focusing imaging (TFM) of the processed data demonstrates that the collaborative detection framework achieves higher signal-to-noise ratios (SNR) compared to single-array imaging. In three parallel experiments, no artifacts with grayscale values exceeding the actual damage were observed, effectively reducing false detection.

The core principle of boundary scattering suppression lies in leveraging the distinct velocity responses of SH_0_ mode guided waves and Lamb waves under frequency-thickness product variations. Specifically, SH_0_ waves maintain constant velocity despite thickness changes (e.g., from the bottom plate to the wall plate), while Lamb waves exhibit velocity dispersion. This disparity introduces asymmetric time-domain characteristics between damage echoes (SH_0_-dominated) and boundary scattering signals (Lamb wave-dominated). The correlation-based processing exploits this asymmetry to selectively attenuate scattering-related wave packets. Consequently, the method achieves optimal performance in T-shaped structures with mismatched bottom plate-wall plate thicknesses, where frequency-thickness product transitions amplify velocity discrepancies. For practical applications, prior knowledge of plate thicknesses is recommended to calibrate the algorithm for specific geometric configurations.

## Figures and Tables

**Table 1:** Material Properties of the T-Joint Finite Element Model for Storage Tank Bottom Plate.

Component	Young’s Modulus *E* (GPa)	Density ρ (kg/m^3^)	Poisson’s Ratio ν
Bottom Plate	208	7850	0.3
Wall Plate	208	7850	0.3
Weld Seam	208	7900	0.3

**Table 2:** Location coordinates and dimensional parameters of defects on the storage tank bottom plate.

Group	Location Coordinates (mm)	Dimensions (mm)	Depth (mm)
1#	$D_{1}$ (1330, 120)	φ = 10	9.3
2#	${D^{\prime }}_{1}$ (1130, 250)	63 × 2	5.3
	${D^{\prime }}_{2}$ (1530, 150)	73 × 2	7.7
3#	${D^{\prime \prime }}_{1}$ (460, 200)	39 × 2	3.1
	${D^{\prime \prime }}_{2}$ (1070, 150)	58 × 2	4.2

**Table 3:** Damage detection results for the storage tank bottom plate.

Actual Damage Location (mm)	Imaging Method	Localization Result (mm)	Relative Error δ	Number of Artifacts with Grayscale Exceeding Damage
$D_{1}$ (1330,120)	Bottom Plate Array Detection	(1380, 90)	3.6%	2
	Collaborative Bottom Plate-Wall Plate Detection	(1264, 115)	5.0%	0
${D^{\prime }}_{1}$ (1130,250)	Bottom Plate Array Detection	(1174, 235)	3.5%	1
	Collaborative Bottom Plate-Wall Plate Detection	(1180, 308)	5.4%	0
${D^{\prime }}_{2}$ (1530,150)	Bottom Plate Array Detection	(1431, 227)	5.8%	0
	Collaborative Bottom Plate-Wall Plate Detection	(1553, 130)	1.4%	0
${D^{\prime \prime }}_{1}$ (460,200)	Bottom Plate Array Detection	(508, 147)	5.4%	1
	Collaborative Bottom Plate-Wall Plate Detection	(450, 196)	2.1%	0
${D^{\prime \prime }}_{2}$ (1070,150)	Bottom Plate Array Detection	(1233, 180)	15.3%	2
	Collaborative Bottom Plate-Wall Plate Detection	(1120, 155)	4.6%	0

## Data Availability

The authors confirm that the data supporting the findings of this study are available from the corresponding author upon reasonable request.
